# Antifungal activity of three different varieties of* Capsicum annuum* against clinical isolates of *Candida* species

**DOI:** 10.1186/s40794-023-00194-w

**Published:** 2023-07-20

**Authors:** Shaghayegh Moghadam, Behnam Azari, Roghayeh Rashidi, Mahdi Hosseini Bafghi, Hassan Rakhshandeh, Selman Mohammed Selman, Hossein Zarrinfar

**Affiliations:** 1 Student Research Committee, Mashhad University of Medical Sciences, Mashhad, Iran; 2 Blood Transfusion Research Center, High Institute for Research and Education in Transfusion Medicine, Tehran, Iran; 3 Neurogenic Inflammation Research Center, Mashhad University of Medical Sciences, Mashhad, Iran; 4 Department of Laboratory Science, Faculty of Paramedical, Mashhad University of Medical Sciences, Mashhad, Iran; 5 Pharmacological Research Center of Medicinal Plants, Mashhad University of Medical Sciences, Mashhad, Iran; 6 Department of Pharmacology, College of Medicine, University of Babylon, Babylon, Iraq; 7 Allergy Research Center, Mashhad University of Medical Sciences, Mashhad, Iran

**Keywords:** *Capsicum annum*, *Candida*, Antifungal

## Abstract

**Objective:**

Acquired resistance to antifungal agents is rising among *Candida* species. Herbal extracts including *Capsicum annum* extracts have biological profits, which can be employed to overcome drug resistance in fungal species. The present study investigated the efficacy of different varieties of *C. annum* extracts against *Candida* species.

**Methods:**

Aqueous and alcoholic extracts of three different varieties of *C. annum* were prepared using the succulent method. Total values for compound extracts of *C. annum var. cayenne, C. annum var. cayenne cultivar sabzevari, and C. annum var. cerasiforme* were 43, 42, and 38 g, respectively. The clinical *Candida* isolates including *C. albicans* (*n* = 13), *C. dubliniensis* (*n* = 2), *C. parapsilosis* (*n* = 2), and *C. tropicalis* (*n* = 1); and reference strains of *C. albicans* (TIMML 1292 and TIMML 183), *C. krusei* (TIMML 1321), *C. parapsilosis* (TIMML 2201), and *C. tropicalis* (TIMML 731) were examined based on the M27-A3 guideline.

**Results:**

Aqueous and alcoholic extracts of *Capsicum annum* showed a minimum inhibitory concentration (MIC) range of more than 512 µg/ml against clinical and reference strains of *Candida*. There was no justifiable difference between the effects of these extracts on *Candida* species.

**Conclusion:**

Both aqueous and alcoholic extracts of *Capsicum annum* could not exert a significant effective impact on clinical and reference strains of *Candida*. The difference in pepper spiciness did not show a significant role against *Candida* isolates. However, their possible effects might be different among other yeasts or filamentous fungi.

## Background

*Candida* species are kind of yeast fungi that are normal flora at the same time causing wide variety of infections from cutaneous to visceral. These Infections have become more frequent and common due to the increase in the use of systemic antibiotics, chemotherapy, corticosteroids, etc. [1–3]. The use of antibiotics disrupts the population of bacteria. This disruption results in an increase in the fungal normal flora population that can be a cause of endogen infection. Most of these infections are caused by *C. albicans*, *C. glabrata*, *C. parapsilosis*, *C. tropicalis*, and *C. krusei* [2, 4]. Nowadays, it is notable that non-*albicans Candida* species have become more opportunistic pathogens in individuals [5]. Multidrug-resistant *Candida* species have become a serious concern, therefore scientists express more interest in natural products such as medicinal plants or essential oils, and their antibacterial or antifungal activities [6, 7]. Herbal extracts and their derivatives have been studied as treatments for bacterial and fungal infections. For example, Curcumin is proven to have antifungal activity against fungal agents [8–10]. Assessing the antimicrobial susceptibility of clinical isolates helps to evaluate the effectiveness of metabolites in these medicinal plants against human pathogens [6, 11]. It is essential to determine the antifungal effects of herbal plants against common invasive *Candida* species since there is not enough evidence about their in vitro activity against *Candida* species*. Capsicum annuum* is cultivated throughout some countries like Iran and Turkey which can be used as a vegetable or condiment [12]. It is known for its high nutritional values such as a wide range of vitamins, minerals, phytochemicals, and dietary fiber, thus it can decrease micronutrient deficiencies [13]. As plants produce pathogenesis-related (PR) proteins against pathogen attack, it is justifiable to assume their antifungal activities [14]. For instance, P14a, P14b, and P14c are isolated from tomato leaves with antifungal effects against *phytophthora infestans* [15]. As a result, both aqueous and alcoholic extracts of *C. annum* were effective against bacteria like *Vibrio cholerae*, *Staphylococcus aureus*, and *Salmonella typhimurium* [12, 16]. However, the alcoholic extract showed greater antibacterial effects than aqueous [16]. Moreover, studies showed *C. annum* as an antioxidant and cytotoxic resource, proving by radical scavenging activity and the MTT assay [17]. Based on the fact that there were no comprehensive antifungal studies on *C. annum* extracts and to overcome drug resistance, this study evaluated the effects of both aqueous and alcoholic extracts of this plant against various *Candida* species.

## Methods

This study was approved by the Ethics Committee (ethics code: IR.MUMS.MEDICAL.REC.1399.454).

The antifungal effects of aqueous and alcoholic extracts of *C. annum* has been evaluated against 18 *Candida* clinical isolates obtained from bronchoalveolar lavage (BAL) specimens of hospitalized children with pulmonary disorders referred to a specialized pediatric Hospital, Mashhad, Iran, including *C. albicans* (*n* = 13), *C. dubliniensis* (*n* = 2), and *C. parapsilosis* (*n* = 2), and *C. tropicalis* (*n* = 1), alongside 5 *Candida* reference strains including *C. albicans* (TIMML 1292, and TIMML 183), *C. krusei* (TIMML 1321), *C. parapsilosis* (TIMML 2201), and *C. tropicalis* (TIMML 731). All clinical isolates were identified by the Vitek MS (bioMérieux, Marcy-L'Etoile, France) and Multiplex PCR [18]. The antifungal susceptibility testing was conducted according to the clinical and laboratory standards institute (CLSI) M27-A3 guidelines. Both aqueous and alcoholic extracts of *C. annum* were prepared referring to the Soxhlet method previously approved by our research group [19]. Peppers were cultivated, collected, and authenticated by Anbari’s agricultural seed company. The herbarium number for each plant was provided as No.13529 for *C. annum var. cayenne,* No.13544 for *C. annum var. cayenne cultivar sabzevari,* and No.13588 for *C. annum var. Cerasiforme*. After transferring fruits to the laboratory, they were dried at room temperature and ground. Fifty grams of each powder was percolated in 1000 ml of 70% ethanol to make a 70% aqueous alcoholic soxhlet extract. It was then placed in the soxhlet apparatus for 72 h. To remove the solvent, the extracts were put in a glass plate on a boiling pan [20]. A part of dried *C. annum* was subjected to solvent–solvent extraction for three fractions, namely, n-butanol fraction (NBF), ethyl acetate fraction (EAF), and aqueous fraction (AQF). In this method, *C. annum var. cayenne, C. annum var. cayenne cultivar sabzevari,* and *C. annum var. Cerasiforme* yielded 43%, 42%, and 38% extracts, respectively. After obtaining the compound extracts, aqueous and alcoholic fractions of them were prepared and kept at -20 °C until use.

**Fig. 1:**
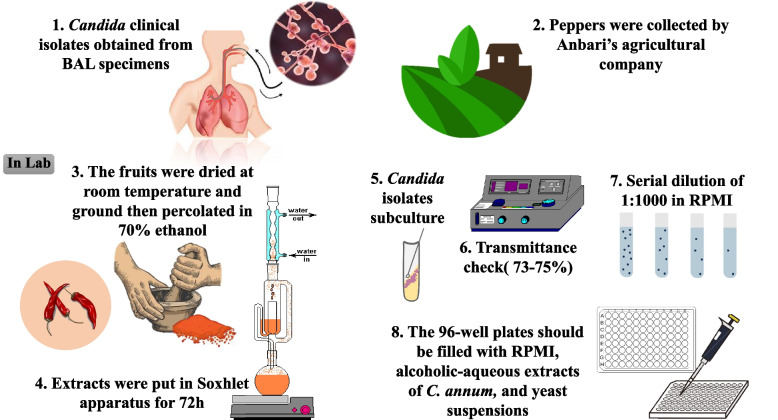
Summary of the steps and procedures performed during the isolation of clinical specimens, and the preparation of *Capsicum annum* extracts and the effect on *Candida* isolates

All *Candida* isolates were sub-cultured on sabouraud dextrose agar (SDA, Merk, Germany) and incubated at 35 °C for 2 days. The inclusion criteria are the colonies of *Candida* that approved by the Vitek MS (bioMérieux, Marcy-L'Etoile, France) and Multiplex PCR [18] and the exclusion criteria are the specimen that haven’t grown in 24-48 h or contaminated by saprophytic fungi. After incubation, the inoculum suspensions were made by dissolving each isolate in a sterile saline solution. Under the CLSI guideline, the transmittance rate of these suspensions was set to be 73–75% at the wavelength of 540 nm using a spectrophotometer. Then the yeast suspensions were diluted 1:1000 in RPMI 1640 medium, containing 3-N-morpholinepropanesulfonic acid (MOPS) (Bio basic, Canada) as a buffer and chloramphenicol as an antibiotic to inhibit bacterial growth. After dilution, the final concentration of each inoculum suspension reached 1–3 × 10^3^ CFU/ml. First, all 96-well plates should be filled with 0.1 ml of RPMI 1640 medium; then the indicated concentrations of both alcoholic and aqueous extracts of *C. annum* along with the yeast suspensions were added to them. These plates were incubated at 35 °C for 2 days. The final concentrations of both aqueous and alcoholic extracts were 1, 2, 4, 8, 16, 32, 64, 128, 256, and 512 µg/ml. Eventually, the minimum inhibitory concentration (MIC) ranges were evaluated visually as the lowest concentration of alcoholic and aqueous extracts, which inhibited at least 80% of the fungal growth in comparison with the positive control, that are included only RPMI and fungal suspension. Moreover, although pungency of pepper extracts was different, pungency did not show a significance efficacy against *Candida* isolates. Figure 1 show the summarized procedures performed during this study.

## Results

Based on the results of this study, none of the compounds, aqueous and alcoholic extracts showed significant fungal growth inhibition, when compared to the positive control. However, among the different variants of *C. annum*, *C. annum var. Cerasiforme* was able to reduce the growth rate of *Candida* species better than other variants used in the study. Although fungal growth was seen in all wells, all three extracts of *C. annum* showed better results against *C. albicans* isolates. As Tables 1 and 2 summarized and provide the data analysis, neither aqueous nor alcoholic extracts of *C. annum* showed any significant MIC (≥ 512 µg/ml) values among clinical isolates and reference strains of *Candida* species. Moreover, although pungency of pepper extracts were different, these differences did not show significant role against *Candida* isolates.

**Table 1:** The antifungal susceptibility profile of aqueous extracts of *Capsicum annum* for clinical isolates and reference strains of *Candida* species

***Candida* species**	**No. (%)**	**Aqueous extracts of *C. annum***	**MIC (µg/ml)**	**Negative Control**	**Positive Control**
*C. albicans*	15 (65.21%)	Variety of* cayenne*	≥ 512	No growth	G
		Variety of *cayenne cultivar sabzevari*	≥ 512	No growth	G
		Variety of *Cerasiforme*	≥ 512	No growth	G
*C. parapsilosis*	3 (13.04%)	Variety of* cayenne*	≥ 512	No growth	G
		Variety of *cayenne cultivar sabzevari*	≥ 512	No growth	G
		Variety of *Cerasiforme*	≥ 512	No growth	G
*C. dubliniensis*	2 (8.69%)	Variety of* cayenne*	≥ 512	No growth	G
		Variety of *cayenne cultivar sabzevari*	≥ 512	No growth	G
		Variety of *Cerasiforme*	≥ 512	No growth	G
*C. tropicalis*	2 (8.69%)	Variety of* cayenne*	≥ 512	No growth	G
		Variety of *cayenne cultivar sabzevari*	≥ 512	No growth	G
		Variety of *Cerasiforme*	≥ 512	No growth	G
*C. krusei*	1 (4.34%)	Variety of* cayenne*	≥ 512	No growth	G
		Variety of *cayenne cultivar sabzevari*	≥ 512	No growth	G
		Variety of *Cerasiforme*	≥ 512	No growth	G
Total	23 (100%)				

MIC: Minimal inhibitory concentration

G: Indicates yeast growth in positive control wells

**Table 2:** The antifungal susceptibility profile of alcoholic extracts of *Capsicum annum* for clinical isolates and reference strains of *Candida* species

***Candida* species**	**No. (%)**	**Alcoholic extracts of *C. annum***	**MIC (µg/ml)**	**Negative Control**	**Positive Control**
*C. albicans*	15 (65.21%)	Variety of* cayenne*	≥ 512	No growth	G
		Variety of *cayenne cultivar sabzevari*	≥ 512	No growth	G
		Variety of *Cerasiforme*	≥ 512	No growth	G
*C. parapsilosis*	3 (13.04%)	Variety of* cayenne*	≥ 512	No growth	G
		Variety of *cayenne cultivar sabzevari*	≥ 512	No growth	G
		Variety of *Cerasiforme*	≥ 512	No growth	G
*C. dubliniensis*	2 (8.69%)	Variety of* cayenne*	≥ 512	No growth	G
		Variety of *cayenne cultivar sabzevari*	≥ 512	No growth	G
		Variety of *Cerasiforme*	≥ 512	No growth	G
*C. tropicalis*	2 (8.69%)	Variety of* cayenne*	≥ 512	No growth	G
		Variety of *cayenne cultivar sabzevari*	≥ 512	No growth	G
		Variety of *Cerasiforme*	≥ 512	No growth	G
*C. krusei*	1 (4.34%)	Variety of* cayenne*	≥ 512	No growth	G
		Variety of *cayenne cultivar sabzevari*	≥ 512	No growth	G
		Variety of *Cerasiforme*	≥ 512	No growth	G
Total	23 (100%)				

MIC: Minimal inhibitory concentration

G: Indicates yeast growth in positive control wells

## Discussion

The incidence of all clinical forms of candidiasis is increasing rapidly, especially among immunocompromised patients [21, 22]. Moreover, the emergence of resistance to conventional antifungal agents urges scientists to expand novel therapeutics against infections caused by *Candida* species. As an example, studies show that fluconazole resistance can be caused by cellular changes induced by stress responses or upregulation of drug transporters [23]. Furthermore, multidrug-resistant species like *C. glabrata* and *C. auris* are becoming more prevalent [24]. Historically, natural products have provided key start-point compounds for therapeutic use. Most of the antimicrobial agents have been traditionally obtained from medicinal plants. *In-vitro* and in vivo antimicrobial activities of herbal extracts and their products such as seeds or fruits have been studied in different regions of the world, which have reported the presence of a wide variety of substances that can restrict the growth of many fungal agents. Based on various studies, some plants or herbal extracts are exhibited to be effective in preventing or curing infectious diseases. Hence, this study has further evaluated the antifungal activity of *Capsicum annum* extracts against clinical and reference isolates of *Candida* species.

Antifungal activities of herbal extracts and medicinal plants were studied overtime against a wide range of fungal agents, such as the antifungal activity of black pepper (*Piper nigrum* leaves) described against *Fusarium oxysporum* and *Aspergillus niger* [25]. The acetone extract of *Piper nigrum* leaves showed an effective impact on the mycelial growth of *F. graminearum*, *Penicillium viridcatum*, and *A. ochraceus* [26]. In 2014, a study reported the antifungal effects of *Pelargonium zonale* stalks against *C. albicans*. The results of this study were based on microscopic analysis, which indicated morphological changes like cell wall damage and deformations of the cell surface [27]. Another research studied the effects of *Astronium urundeuva* leaves and demonstrated the antifungal activity by using its free extract and microemulsion against *C. glabrata* and *C. albicans* [28]*.* Based on a study conducted by Prabhakar et al*.*, alcoholic extracts of *Syzygium jambolanum, Cassia siamea, Caulerpa scalpelliformis,* and *Sargassum wightii* showed significant antifungal activity against *Candida* species isolated from oral lesions [29]. In another study in Saudi Arabia, researchers evaluated the antifungal activity of a traditional plant called *Myrtus communis* against *Candida* species; the results showed that its root and leaf extracts had acceptable antifungal activity against *C. glabrata* through damaging the cell membrane [30]. In 2018, Jameel et al*.* studied the antifungal activities of methanolic, hexane, and aqueous extracts of *Capparis deciduas* against *C. albicans* and concluded that the methanolic extract had a more significant inhibitory effect against the fungal species used in the study [31]. Wenji et al. researched to determine about the antifungal activity of Peppermint (*Mentha piperita* leaves*)*, which showed a high inhibitory activity of Mint leaf extracts against *C. albicans* [32]. In 2015, *Aloe vera* extracts were assessed and showed considerable antifungal activity against *C. albicans* [33]. Studies by Sytykiewicz et al*.* concluded that methanolic extracts of walnut leaves have the highest antifungal activity against *C. albicans,* while its ethyl acetate and hydrolyzed methanolic extracts exhibit lower inhibitory effects against *C. albicans’* growth rate [34]. On the other hand, the features of sweet pepper (*C. annum* leaves*)* have shown that it is best known as a source of Vitamins C and E [17]. Currently, in India, America, and China, *C. annum* is being used as a treatment for arthritis, rheumatism, dog/snake bites, and flesh wounds [17]. *C. annum* has different genotypes and seasonal breeding and grows in different shapes, sizes, and colors, which can affect its antifungal activity [17]. Many studies were conducted and showed that *C. annum* has potential antibacterial and antioxidant activities. Moreover, it can positively affect human breast, prostate, and cervical tumors [17]. Although in some studies the effectiveness of *Capsicum* extracts was not uniform, there is strong evidence about the antibacterial effects of these extracts against *S. aureus*, *S. typhimurium*, and *V. cholerae* [16]. According to a study in 2012, *C. annuum var. Antillais* and *C. Frutescens var. Soudanese* showed great antibacterial effects against *V. cholerae* [16]. As of today, there is no conclusive research about the antifungal effects of *C. annuum*, therefore this study was conducted as an attempt to discover whether *C. annuum*, as an antifungal agent, has any impact on *Candida* isolates or not. Since some plant extracts have specific active substances that attack different structures of fungal agents, in this case, these active substances in pepper are probably weak or ineffective against wall structures, cytoplasmic membrane and proteins. Therefore although the current study yielded no significant results, more studies should be done due to the lack of knowledge in this field.

## Conclusion

The results of the present study showed that none of the compound, aqueous and alcoholic extracts of *C. annuum* had any significant inhibitory effects against clinical and reference strains of *Candida*. Although the pungency of pepper extracts were different, these differences did not show a significant role against *Candida* isolates. The present study has some limitations, including the insufficient number of various species of *Candida*. Additionally, a small size population of clinical isolates was not included. Thus, further investigations are required to determine whether *C. annuum* or its active components, such as capsaicin, capsaicinoids, and carotenoids, have any other antifungal activity against *Candida* spp. or other fungal pathogens.

### Indexing purposes

Based on the fact that there were no comprehensive antifungal studies on *C. annum* extracts, this study evaluated the effects of both aqueous and alcoholic extracts of this plant against various *Candida* species. Moreover, due to the emergence of antifungal drug resistance can be a crucial issue.

## Data Availability

The datasets used and/or analyzed during the current study are available from the corresponding author on reasonable request.

## Abbreviations

CLSI: Clinical and laboratory standards institute
BAL: bronchoalveolar lavages
GM: geometric mean
